# Biological Effects of Tetrahydroxystilbene Glucoside: An Active Component of a Rhizome Extracted from *Polygonum multiflorum*

**DOI:** 10.1155/2018/3641960

**Published:** 2018-11-04

**Authors:** Lingling Zhang, Jianzong Chen

**Affiliations:** ^1^Translational Medicine Center, Honghui Hospital, Xi'an Jiaotong University, Xi'an 710054, China; ^2^Traditional Chinese Medicine Department, Xijing Hospital, Fourth Military Medical University, Xi'an 710032, China

## Abstract

*Polygonum multiflorum* Thunb. (PM), a traditional Chinese medicinal herb, has been widely used in the Orient as a tonic and antiaging agent. 2,3,5,4′-Tetrahydroxystilbene-2-*O*-*β*-D-glucoside (TSG, C_20_H_22_O_9_, FW = 406.38928) is one of the active components extracted from PM. TSG is an antioxidant agent, which exhibits remarkable antioxidative activities in vivo and in vitro. The antioxidant effect of TSG is achieved by its radical-scavenging effects. TSG can inhibit apoptosis and protect neuronal cells against injury through multifunctional cytoprotective pathways. TSG performs prophylactic and therapeutic activities against Alzheimer's disease, Parkinson's disease, and cerebral ischemia/reperfusion injury. It is also antiatherosclerotic and anti-inflammatory. However, the mechanisms underlying these pharmacological activities are unclear. This study aimed at reviewing experimental studies and describing the effectiveness and possible mechanisms of TSG.

## 1. Introduction

The root of *Polygonum multiflorum* Thunb. (PM), which is also known as heshouwu, is a famous traditional Chinese medicinal herb that has been used as a tonic and antiaging agent in the Orient. PM and its extract can be used to treat age-related diseases [1–3]. The medicinal effects of PM in the treatment of these age-related diseases are possibly mediated by the antioxidant capacity of this plant [4], because free radical-induced oxidative stress has been implicated in the aging process. PM consists of anthraquinone, stilbene, phospholipid, and other compounds, and modern chromatographic separation studies have demonstrated that many bioactive compounds (e.g., stilbene glycosides) in PM are responsible for its medicinal activities [5]. One of the main components that can be extracted from the root of PM is 2,3,5,4′-tetrahydroxystilbene-2-*O*-*β*-D-glucoside (TSG), which is a stilbene monomer [6]. Since the discovery of TSG by Hata et al. [7], who were Japanese scientists, the chemical components of PM have been widely explored in academic medicine. The pharmacological characteristics of the compound have also been investigated. TSG possesses special biological actions and considerable values in scientific research and clinical medicine. The structure of TSG is similar to that of resveratrol (3,4′,5-trihydroxy-*trans*-stilbene), which is well known for its numerous biological activities, especially in cardiovascular protection and neuroprotection. TSG possesses strong antioxidant and free radical-scavenging activities, which are much stronger than those of resveratrol in superoxide anion radical scavenging, hydroxyl radical scavenging, and DPPH radical scavenging [8]. Parkinson's disease (PD) is among the most common age-related neurodegenerative disease. Current pharmacological treatments of PD remain largely symptomatic, and the development of new therapeutic strategies may provide effective alternative treatment options. Vergara et al. [9] provided experimental evidence supporting the beneficial effects of resveratrol in preserving cellular homeostasis in parkin-mutant fibroblasts, which may be relevant for PD treatment. The polyphenolic structure of TSG is similar to that of resveratrol. TSG also elicits neuroprotective effects in various neurodegenerative diseases and cerebral ischemia. In aged mice, TSG treatment rescues synapses and suppresses *α*-synuclein overexpression in the brain, subsequently improving their memory and movement functions [10]. TSG protects dopamine (DA) neurons against lipopolysaccharide- (LPS-) induced neurotoxicity through dual modulation on glial cells by attenuating microglial-mediated neuroinflammation and enhancing astroglial-derived neurotrophic effects [11]. In an ischemia-reperfusion-injured rat model, TSG promotes the postoperative recovery of rats by minimizing the volume of cerebral infarcts and improving neurological dysfunction, thereby upregulating the expression levels of CD31, angiopoietin 1, and angiopoietin receptor-2 [12]. TSG possesses antiatherosclerosis, anti-inflammatory, and anticardiac fibrotic effects. Although these effects are widely known, the mechanisms of action have yet to be established. Numerous mechanisms, such as activation of adenosine 5′-monophosphate-activated protein kinase [13, 14], protein kinase B (Akt) [15, 16], peroxisome proliferator-activated receptor gamma (PPAR-*γ*) [17], and silent mating type information regulation 2 homolog 1 [18] and inhibition of the classical nuclear factor-*κ*B (NF-*κ*B) signal [13], mediate the therapeutic effects of TSG. This study aimed at reviewing various experimental studies and describing the effectiveness and possible mechanisms of TSG.

## 2. Antioxidative Effect

Oxidative stress is a phenomenon that induces various health disturbances and diseases by enhancing the oxidation of biologically important molecules in vivo. Oxidation reactions by reactive oxygen species (ROS) are regarded as factors that trigger oxidative stress [19]. They are a group of very reactive short-lived chemicals produced during normal metabolism or after an oxidative reaction. ROS include free radicals, such as superoxide anion (O_2_^·−^) and hydroxyl radical (^·^OH), and nonradical molecules, such as hydrogen peroxide (H_2_O_2_) and singlet oxygen (^1^O_2_) [20]. Under physiological conditions, an appropriate level of intracellular ROS should be maintained to achieve redox balance and cell proliferation [21]. However, excessive ROS accumulation is highly cytotoxic because these chemicals induce DNA damage, lipid peroxidation, and protein degradation [20, 22]. Oxidative damages caused by ROS may cause various neurodegenerative and chronic diseases, such as coronary heart diseases, atherosclerosis, cancer, and aging [23]. DPPH is a free radical widely used to test the free radical-scavenging ability of various chemicals. TSG possesses a strong DPPH radical-scavenging activity in vitro with an IC_50_ of 33.24 *μ*Μ [24]. TSG also exhibits a strong capacity to scavenge ^·^OH and superoxide anion radical with IC_50_ of 2.75 and 0.57 *μ*g/ml, respectively [8]. The crucial components of an antioxidant defense system in the body are cellular antioxidant enzymes (e.g., superoxide dismutase (SOD) and glutathione peroxidase (GSH-Px)), which are involved in the reduction of ROS and peroxides produced in living organisms and in the detoxification of certain exogenous compounds. In human brain microvascular endothelial cells, TSG exhibits cytoprotective effects against H_2_O_2_-induced oxidative stress by inhibiting malondialdehyde (MDA) and ROS and upregulating SOD and GSH [25]. To further evaluate the in vivo antioxidative effects of TSG on rats, Lv et al. [26] administered D-galactose (100 mg/kg/day body weight; single hypodermic injection) to senile rats and applied TSG (20 and 40 mg/kg/day body weight). They found an increase in the activities of SOD and GSH-Px and a decrease in the content of thiobarbituric acid reactive species in the serum and heart, brain, and liver tissues of rats. TSG also reduces serum MDA and tissue 8-OHDG levels and increases serum GSH-Px activity in hypertensive rats aged spontaneously [27]. Hemeoxygenase-1 (HO-1) is a highly inducible and stress-responsive protein (also called heat shock protein 32) that catalyzes the first and rate-limiting step in the degradation of heme, which is a potent oxidant [28]. NADPH-quinone oxidoreductase 1 (NQO1) is a predominantly cytosolic enzyme that provides cells with multiple layers of protection against oxidative stress, including the direct detoxification of highly reactive quinones [29]. The pretreatment of cells with TSG (5 and 10 *μ*M) for 24 h can attenuate H_2_O_2_-induced ROS production and upregulate the expression of antioxidant enzymes HO-1 and NQO1 in a dose-dependent manner [30]. These results suggest that TSG is a potent antioxidant.

## 3. Neuroprotective Effect

### 3.1. Protective Effect against Alzheimer's Disease

Alzheimer's disease (AD) is a neurodegenerative disorder characterized by a progressive decline in cognitive abilities and associated with neuropathological features, including extensive loss of neurons (particularly cholinergic neurons), intracellular neurofibrillary tangles composed of the tau protein, and extracellular deposition of plaques composed of *β*-amyloid (A*β*), which is a cleavage product of amyloid precursor protein (APP). These two insoluble protein aggregates are accompanied by chronic inflammatory responses and extensive oxidative damage [31]. Learning and memory abilities are important cognition aspects that reflect the advanced integrative functions of the brain. Modern research has shown that an impaired memory function may be associated with cholinergic system synthases, such as acetylcholine transferase (ChAT) and hydrolytic enzymes, such as acetylcholinesterase (AChE). The levels of ChAT and acetylcholine (Ach) decrease in patients with AD, whereas the concentration of AChE increases [32]. Learning and memory abilities are closely related to central nervous system function, and their regulation involves monoamine neurotransmission; NE, DA, and 5-HT levels change with age [33]. Senescence-accelerated-prone 8 (SAMP8) mice exhibit early onset impaired learning and memory [34]. TSG significantly improves memory ability (*P* < 0.01) compared with that of the control group and prolongs the life span of SAMP8 mice by 17% (*P* < 0.01) compared with that of the control group [3]. TSG increases the protein level of neural klotho and reduces the levels of neural insulin, insulin receptor, insulin-like growth factor-1 (IGF-1), and IGF-1 receptor in the brain of SAMP8 mice (*P* < 0.01) compared with that of the control group [3]. In a hidden platform test and a spatial probe test, age-related learning and memory impairment in SAMP8 mice is prevented by daily treatment with TSG (33, 100, and 300 mg/kg/day) for 50 days [35]. In the same manner, TSG induces a dose-dependent enhancement of the activity of ChAT, decreases the activity of AChE, promotes the synthesis of monoamine neurotransmitter (NE, DA, and 5-HT), downregulates glutamic acid and aspartic acid, and retrieves the metabolic disorder of amino acid neurotransmitters in the brain tissues of SAMP8 mice compared with those of the SAMP8 group (*P* < 0.05 or 0.01) [36, 37].

A*β*, a major protein component of senile plaques, is considered as a critical cause of the pathogenesis of AD [31]. The alternative splicing of APP exon 7 generates isoforms containing a Kunitz protease inhibitor (KPI) domain. APP–KPI levels in the brain are correlated with A*β* production [38]. TSG protects nerve cells against A*β*-induced cell damage and improves learning–memory deficit in an AD model of APP transgenic mice and in a rat model of cholinergic damage induced by injecting ibotenic acid into the basal forebrain [39, 40]. Moreover, intragastric TSG (100 mg/kg/day) administration for 1 month can significantly improve learning, memory, spatial orientation, and other behavioral functions in transgenic mice and attenuate A*β* neurotoxicity-induced injury to endoplasmic reticulum functions [41]. In cell lines and APP_SWE_/PSEN_dE9_ (APP/PS1) transgenic mice, TSG suppresses APP–KPI^+^ and amyloid plaque formation. The mechanism of the neuroprotective effect of TSG may involve the activation of the AKT–GSK3*β* signaling pathway, the attenuation of the splicing activity of ASF, and the decrease in APP–KPI^+^ levels, leading to the decline in A*β* deposition [15]. In addition, the intragastrical administration of 60 and 120 mg/kg TSG increases mitochondrial COX activities; decreases A*β*_1–42_ contents; reduces APP, beta-site APP cleaving enzyme 1, and presenilin-1 expression; and enhances nerve growth factor, brain-derived neurotrophic factor, and tropomyosin-related kinase B expression in the hippocampus of NaN_3_-infused rats [42]. A*β* deposition-induced microglial activation is a crucial event in the pathology of AD [43]. Jiao et al. [44] found that TSG attenuates A*β*-induced microglial activation and inhibits the production of inflammatory molecules, such as inducible nitric oxide synthase (iNOS), nitric oxide (NO), cyclooxygenase 2 (COX-2), and prostaglandin E2. Furthermore, A*β* exposure increases the levels of microglial M1 markers, interleukin- (IL-) 1*β*, IL-6, and tumor necrosis factor *α* (TNF-*α*). TSG pretreatment suppresses the increase in M1 markers and enhances the levels of M2 markers, such as IL-10, brain-derived neurotrophic factor, glial cell-derived neurotrophic factor, and arginase-1, in N9 and BV2 cells. PU.1 overexpression partly eliminates the anti-inflammatory effects of TSG, suggesting that the roles of TSG in A*β*-induced microglial cells are mediated by PU.1 expression.

Oxidative damage in macromolecules is associated with the accumulation of A*β* in the progressive development of AD [45] and is a critical event in A*β*-induced neuronal cell death [46]. In vitro models, TSG prevents A*β*_1–42_-induced PC12 cell injury by improving the cell survival rate, reduces LDH release and MDA content, and increases SOD activity and Bcl-2 expression [47]. In vivo, the intragastric administration of TSG (100 mg/kg/day) in drinking water for 8 weeks provides protection against memory impairment induced by intracerebroventricular treatment with A*β*_1–40_ (3 *μ*g/mouse, i.c.v.) in mice. In addition, TSG reduces interleukin-6 (IL-6) content (*P* < 0.05), MDA content (*P* < 0.01), and MAO-B activity (*P* < 0.05) and increases T-AOC activity (*P* < 0.01) compared with those of the model group [48, 49]. Other studies have shown that TSG potentially reverses alterations in cognitive behavioral, biochemical changes, and oxidative damage induced by A*β*_1–42_ in mice. These beneficial effects of TSG can be attributed partly by inhibiting the expression of the Keap1/Nrf2 pathway in hippocampus and cerebral cortex tissues [50]. Some investigations have also suggested that TSG protects neuronal HT-22 cells against A*β*-induced neurocytotoxicity by ameliorating mitochondrial-dependent oxidative stress and apoptotic pathway via the activation of Nrf2-HO-1 signaling. These data may elucidate a new mechanism about how TSG attenuates the pathologic process of AD by repairing A*β*-induced hippocampal neuron impairment [51].

A*β* enhances the oligomerization, accumulation, and toxicity of *α*-synuclein [52]. *α*-Synuclein is a highly abundant protein at presynaptic terminals, is associated with the distal reserve pool of synaptic vesicles, and has a role in the regulation of neurotransmitter release, synaptic function, and plasticity [53]. Synaptic plasticity in the hippocampus has been considered the key phenomenon of learning and memory processes [54]. A previous study demonstrated that 120 and 240 *μ*mol/kg/day TSG improves learning–memory impairment, decreases A*β* content, and inhibits *α*-synuclein overexpression and aggregation in the hippocampus of APPV717I transgenic mice in an AD model [40, 55].

The intragastric administration of 60 mg/kg/day TSG to 21-month-old rats for 3 months remarkably improves their learning–memory abilities in water maze tests, increases the number of synapses and synaptic vesicles, and increases the expression of synaptophysin in the hippocampal CA1 region of aged rats [56]. Another study has demonstrated that the oral administration of TSG for 3 months enhances memory and movement functions, protects the synaptic ultrastructure, and increases the synaptic connections and the levels of synapse-related proteins and p-CaMKII in the hippocampus, striatum, and cerebral cortex of aged mice [10]. The long-term potentiation (LTP) of synaptic transmission triggered by high-frequency stimulation (HFS) in the hippocampal CA1 area requires postsynaptic molecular mechanisms, such as the activation of *N*-methyl-D-aspartate (NMDA) receptors, calcium–calmodulin-dependent protein kinase II (CaMKII), and extracellular signal-regulated kinases (ERKs) of the mitogen-activated protein family [57–60]. In vitro, 1 and 5 *μ*M TSG induce neurite outgrowth, promote PC12 cell differentiation, and increase intracellular calcium levels in hippocampal neurons in a concentration-dependent manner. Moreover, TSG facilitates HFS-induced hippocampal LTP in a bell-shaped manner. The facilitation of LTP induction by TSG requires CaMKII and ERK activation [61]. Therefore, TSG can be used to treat Alzheimer's disease.

### 3.2. Protective Effect against Parkinson's Disease

Parkinson's disease (PD) is the second-most common neurodegenerative disorder caused by the loss of dopaminergic (DA) neurons in the substantia nigra pars compacta (SNpc) and the presence of ubiquitinated alpha-synuclein- (*α*-syn-) containing cytoplasmic inclusions called Lewy bodies in surviving SNpc neurons [62]. Several factors, including oxidative stress, mitochondrial dysfunction, neuroinflammation, and dysregulated kinase signaling, likely operate in the mechanism of death of nigrostriatal DA neurons in PD [63–65]. Many studies have used 1-methyl-4-phenyl-1,2,3,6-tetrahydropyridine (MPTP) or its active metabolite 1-methyl-4-phenylpyridinium (MPP^+^), 6-hydroxydopamine (6-OHDA) and paraquat (PQ) as neurotoxins or Parkinsonism mimetics in cell cultures or animal PD models. These toxins induce oxidative stress and lead to cell death of DA neurons to mimic the situation in PD [66].

Oxidative stress is an important factor that can modulate intracellular signaling and lead to neuronal death by apoptosis or necrosis [67, 68]. These include JNK signaling, p38 activation, PI3K/Akt signaling inactivation, and signaling through bcl-2 family proteins [65]. MTT assay, flow cytometry, and DNA fragmentation have confirmed that TSG elicits protective effects against MPP^+^-induced PC12 cell apoptosis by inhibiting ROS generation and modulating the activation of JNK and the PI3K/Akt pathway [69, 70]. TSG remarkably enhances the antioxidant enzyme activities of SOD, catalase (CAT), and GSH-Px and efficiently reduces the MDA content in PC12 cells [71]. MPP^+^ transports into DA neurons and accumulates in the mitochondria, resulting in ATP depletion and mitochondrial membrane potential alteration [72]. TSG inhibits the increase in intracellular reactive oxygen species levels and the MPP^+^-induced disruption of mitochondrial membrane potential. TSG markedly upregulates the Bcl-2/Bax ratio, reverses the cytochrome c release, and inhibits the caspase-3 activation in MPP^+^-induced PC12 cells or SH-SY5Y cells [71, 73]. In vitro studies have shown that MPP^+^ increases *α*-Syn expression [74], and the overexpression of mutant human *α*-Syn aggravates MPP^+^-induced neurotoxicity [75]. TSG (3.125–50 *μ*M) protects A53T AS cells against MPP^+^-induced cell damage, and the mechanisms of TSG's neuroprotective effects are mediated by inhibiting *α*-Syn overexpression and aggregation, enhancing mitochondrial function, reducing ROS levels, and inhibiting apoptosis in A53T AS cells exposed to MPP^+^ [76]. NO is involved in the pathogenesis of PD [77]. NOS has four known isoforms: nNOS, iNOS, eNOS, and mitochondrial NO synthase. nNOS and iNOS are closely related to the pathogenesis of PD [78]. Our study showed that the exposure of PC12 cells to 75 mM 6-OHDA for 24 h significantly increases the level of intracellular ROS and NO, induces the overexpression of iNOS and nNOS, and elevates the level of 3-NT. However, these changes are markedly reversed in a dose-dependent manner after PC12 cells are pretreated with different TSG concentrations for 24 h. These results suggest that TSG may protect PC12 cells from 6-OHDA-induced apoptosis by regulating the ROS–NO pathway [79].

To further evaluate the in vivo neuroprotective effect of TSG, Zhang et al. and He et al. [16, 80] treated mice with TSG (20 or 40 mg/kg, i.g.) for 14 days, conducted pole and open field tests, and found that this treatment significantly reverses the MPTP-induced behavioral deficits compared with those of the MPTP treatment group (*P* < 0.05). Behavioral disorders occur when the number of TH-positive neurons in the SNpc of MPTP-treated mice decreases to 57.04% of the normal amount [81]. In another study, mice injected with MPTP show an approximately 63% decrease in TH neurons and manifest typical symptoms and behavioral disorders, such as tremor, piloerection, and bradykinesia. Conversely, mice treated with TSG exhibit a significant improvement in behavior and TH-positive neurons, which recover to 66% of that in the control group [80]. DA and its metabolites (DOPAC and HVA) decrease after MPTP is injected in striatal neurons. TSG dose-dependently counteracts the MPTP-induced loss of striatal DA [80], prevents the loss of striatal DA transporter protein [16], and provides protection against MPTP lesions partly by controlling ROS-mediated JNK, P38, and mitochondrial pathways and PI3K/Akt-mediated signaling mechanism. These in vivo effects extend previous in vitro observations [70, 71].

Neuroinflammation is an important contributor to PD pathogenesis with the hallmark of microglial activation [82]. Daily intraperitoneal injection of TSG for 14 consecutive days significantly protects DA neurons from 6-OHDA-induced neurotoxicity and suppresses microglial activation. A similar neuroprotection is shown in primary neuron–glial cocultures. In vitro studies have further demonstrated that TSG inhibits the activation of microglia and the subsequent release of proinflammatory factors. Moreover, TSG-mediated neuroprotection is closely related to the inactivation of mitogen-activated protein kinase signaling pathway [83]. Astroglia also plays an important role in PD development and becomes the prime target of PD treatment [84]. TSG protects DA neurons against LPS-induced neurotoxicity through dual modulation on glial cells by attenuating microglial-mediated neuroinflammation and enhancing astroglial-derived neurotrophic effects. These findings may serve as a basis for developing new alternative PD treatments [11].

### 3.3. Protective Effects against Cerebral Ischemia Injury

Multiple pathogenic mechanisms are involved in ischemia/reperfusion- (I/R-) related injury, including oxidative stress, inflammation, excitotoxicity, calcium overload, and apoptosis [85, 86]. ROS have been considered important mediators of brain damage after I/R injury [86, 87]. In vitro, various TSG concentrations (25, 50, and 100 *μ*M) have been reported to protect the primary culture of cortical neurons against cytotoxicity induced by oxygen–glucose deprivation followed by reperfusion (OGD-R). Moreover, TSG (25 *μ*M) reverses OGD-R-induced neuronal injury, intracellular ROS generation, and mitochondrial membrane potential dissipation and attenuates H_2_O_2_-induced increase in [Ca^2+^]_i_ [18]. Xu et al. [88] showed an ameliorating effect of TSG against focal cerebral I/R injury-induced apoptosis that can be attributed to its antioxidative actions. Doses of 0.038, 0.114, and 0.342 g/kg/day of TSG administered intragastrically at the onset of I/R in rats for 7 days result in a significant increase in GSH-Px and SOD activities (*P* < 0.05) but a decrease in the MDA content (*P* < 0.01) [88]. In another study that involves TUNEL staining, the intraperitoneal administration of TSG at dosages of 15 and 40 mg/kg at the onset of reperfusion after 90 min of middle cerebral artery occlusion (MCAO) in mice causes significant reductions in the brain infarct volume and the number of positive cells in the cerebral cortex compared with those of the MCAO group [18]. In addition, the continuous intragastric administration of TSG to mice for 6 days before I/R relieves the increase in the binding force of NMDA receptor (*P* < 0.05). Optical section results have shown that the calcium concentration in the group treated with TSG is remarkably lower than that in the ischemia model group (*P* < 0.05 or 0.01) [89]. A decrease in cellular oxygen tension during ischemic stroke also promotes an endogenous adaptive response accompanied by the upregulation of various cytoprotective factors to attenuate ischemic injury. The cytokines erythropoietin (EPO) and insulin-like growth factor I (IGF-I) are individually found to be effective [90]. The administration of TSG (60 mg/kg/d) can improve the neurological function of rats after reperfusion and increase the protein expression of HIF-1*α* and EPO after reperfusion compared with those of the model group [91]. In a related paper by Zhao et al. [92], TSG (30, 60, and 120 mg/kg/d) taken orally for 7 days can inhibit cell apoptosis caused by focal cerebral ischemia in rats, and its mechanism may be involved in the increase in the expression of Bcl-2 protein, which can inhibit cell apoptosis, and in the decrease in the expression of Bax protein, which can induce cell apoptosis and decrease the ratio of Bcl-2/Bax.

Neuronal death that follows 10 min of ischemia is associated with a late increase in AChE activity [93]. Microtubule-associated protein-2 (MAP-2) is depleted in the early hours after an in vivo ischemia insult in a rat hippocampal slice [94]. Experiments involving rats with chronic cerebral ischemia have demonstrated that the administration of TSG (30, 60, and 120 mg/kg) for 11 weeks causes a dose-dependent decrease in the AChE activity and increases the expression of protein phosphatase-2A (PP-2A) and MAP-2 in the hippocampus [95]. Angiogenesis is a prognostic marker of the survival and functional improvement of patients with cerebral stroke. Intraperitoneal injection of TSG (30, 60, and 120 mg/kg) promotes postoperative recovery in rats by minimizing the volume of cerebral infarcts and improving neurological dysfunction in a dose- and time-dependent manner. Additionally, TSG significantly increases the microvessel density in the brain and upregulates the CD31 expression in the ischemic penumbra relative to that in the control. Finally, treatment with TSG significantly upregulates the relative levels of vascular endothelial growth factor, angiopoietin 1, and angiopoietin receptor-2 expression in the brain lesions of rats [12]. Thus, TSG elicits neuroprotective effects against cerebral ischemia injury by modulating various pathways.

## 4. Antiatherosclerosis Effect

Atherosclerosis is a chronic and progressive disease in which plaques consisting of deposits of cholesterol and other lipids, calcium, and large inflammatory cells called macrophages build up in arterial walls. Although the pathophysiological mechanisms underlying atherosclerosis are poorly understood, inflammation and oxidative stress play important roles in all the phases of atherosclerosis evolution [96]. ROS can promote inflammation, alter vasomotion, induce cell death, cause platelet aggregation, and stimulate vascular smooth muscle cell (VSMC) proliferation [97]. One of its main risk factors is low-density lipoprotein (LDL) cholesterol [98]. When LDL penetrates endothelial cells, more ROS are generated. In turn, ROS oxidize LDL to oxidative low-density lipoprotein (OX-LDL), which can cause endothelial injury and inflammatory reaction [99]. All these events contribute to cardiovascular lesion formation.

TSG inhibits atherosclerosis by regulating blood fat, antioxidant and anti-inflammatory effects, suppressing matrix metalloproteinase expression, and relaxing blood vessels [100–103]. Gao et al. [100] reported that the administration of TSG (90 and 180 mg/kg/day) to hyperlipidemic rats for 1 week significantly reduces atherosclerosis index (AI, LDL-C/HDL-C) and serum total cholesterol (TC) and low-density lipoprotein cholesterol (LDL-C) levels (*P* < 0.01) but increases the mRNA expression of LDL receptor (LDLR) (*P* < 0.05) in liver cells. In another study, after rats are orally administered with 12 and 24 g/kg of TSG and a high-fat diet for 4 weeks, the contents of TC, TG, LDL-c, apoB, and MDA decrease significantly. The energy of serum SOD, CAT, GSH-Px, and T-AOC and the ratios of HDL-c/TC and apoAI/apoB also significantly increase in TSG groups, suggesting that TSG can regulate lipid metabolism and elicit an antioxidant effect [101]. ApoE^−/−^ mouse is the first mouse model to develop lesions similar to those of humans and mimic the initiation and progression of human atherosclerosis. After 8 weeks of treatment, TSG ameliorates the serum levels of TC, TG, and LDL-C and increases the serum level of high-density lipoprotein cholesterol in ApoE^−/−^ mice. TSG suppresses hepatic steatosis, atherosclerotic lesion formation, and macrophage foam cell formation in ApoE^−/−^ mice. Moreover, TSG improves the expression levels of hepatic scavenger receptor class B type I (SR-BI), ABCG5, and CYP7A1 and upregulates the protein expression levels of aortic ATP-binding cassette transporters G1 and A1 (ABCG1 and ABCA1). An in vitro study has shown that TSG promotes the cholesterol efflux of macrophages and increases the protein expression levels of ABCA1 and ABCG1 [104].

Endothelial dysfunction is a key early event in atherosclerotic plaque formation and characterized by inflammatory processes, reduced NO bioavailability, and increased oxidative stress [105, 106]. TSG can prevent the development of atherosclerosis by influencing endothelial function in atherogenic diet-fed rats [103]. The administration of TSG (30, 60, and 120 mg/kg/day) after atherosclerosis is induced for 6 weeks results in a dose-dependent increase in the NO levels in the serum and aorta, the NOS content in the aorta, and the expression of eNOS but reduces the expression of iNOS in the aorta of atherosclerotic rats [107]. In the same manner, TSG improves ACh-induced endothelium-dependent relaxation, prevents intimal remodeling, and inhibits the decreased NOx content in the serum and aorta of atherogenic diet-fed rats after 12 weeks of treatment. The decreased mRNA and protein expression of eNOS and the increased mRNA and protein expression of iNOS in atherogenic diet-fed rats are attenuated by TSG treatment. These results suggest that TSG can restore vascular endothelial function, which may be related to its ability to prevent changes in eNOS and iNOS expression, leading to the preservation of NO bioactivity [103].

Inflammatory processes also play important roles in the onset, development, and remodeling of atherosclerotic lesions [108]. C-reactive protein (CRP) is probably a mediator of atherosclerosis and may increase the vulnerability of an atherosclerotic plaque to rupture [109]. After adhering to and migrating into the vascular wall at atherosclerotic sites, leukocytes secrete proinflammatory cytokines, such as IL-6, which likely play a major role in the pathogenesis of atherosclerosis [110]. Tumor necrosis factor- (TNF-) *α*, another cytokine with proinflammatory effects, is upregulated in atherosclerotic plaques and may contribute to the pathogenesis of atherosclerosis [111]. Different doses of TSG (120, 60, or 30 mg/kg/day) administered to Sprague–Dawley rats for 12 weeks significantly and dose-dependently attenuate hyperlipidemic diet-induced alterations in serum lipid profile and increase CRP, IL-6, and TNF-*α* levels [102]. Zhao et al. [112, 113] showed that TSG inhibits lysophosphatidylcholine- (LPC-) induced apoptosis of human umbilical vein endothelial cells (HUVECs) by blocking the mitochondrial apoptotic pathway, and this process is accompanied with the activation of superoxide dismutase and glutathione peroxidase, the clearance of intracellular reactive oxygen species, and the reduction of lipid peroxidation. In addition, TSG provides protection against LPC-induced endothelial inflammatory damage. This protective effect is indicated by improved cell viability, adhesive ability, and migratory ability. Moreover, TSG reduces the expression and prevents the LPC-enhanced expression of Notch1, Hes1, and MCP-1. Therefore, the protective effects of TSG against inflammatory damage partly depends on Notch1 inhibition.

VSMCs are the main cellular components in the blood vessel wall, and their excessive proliferation plays an important role in the pathogenesis of atherosclerosis [114]. Although several growth factors and cytokines are involved in the development of atherosclerotic lesions, one of the principal regulators of mitogenesis in VSMCs is platelet-derived growth factor- (PDGF-) BB whose expression is increased in atherosclerotic lesions. PDGF-BB has been shown to activate key extracellular signaling transducers, including ERK 1/2, which are associated with cell growth and movement and critical for the initiation and progression of vascular lesions [115]. Xu et al. [116] demonstrated that TSG (10 and 100 *μ*mol/l) significantly inhibits VSMC proliferation induced in the serum, cell cycle transition from the G_0_/G_1_ phase to the S phase, and proliferating cell nuclear antigen expression in the nucleus of VSMCs. Vimentin is a cytoskeletal protein involved in VSMC proliferation, monocyte migration across the endothelium walls, and foam cell formation [117]. Yao et al. [118, 119] reported that vimentin is a key protein in the TSG treatment for atherosclerosis in rats, and TSG attenuates vimentin mRNA and protein levels in oxLDL-induced HUVECs. This protective effect may be mediated partly by TSG-induced inhibition of vimentin expression via the interruption of the TGF*β*/Smad signaling pathway and caspase-3 activation. These findings help elucidate the molecular mechanism underlying the beneficial effects of TSG on atherosclerosis and suggest that TSG may be an effective agent for cardiovascular disease.

## 5. Effects on Inflammatory Disease

NF-*κ*B is a transcription factor that promotes the transcription of genes involved in proinflammatory responses [120]. The activation of NF-*κ*B leads to the formation of NF-*κ*B dimers (p65 and p50) that translocate to the nucleus to promote the transcription of the proinflammatory mediators TNF-*α*, IL-6, and COX-2, resulting in a series of inflammatory cascade responses [121]. PPAR-*γ*, a member of the nuclear hormone receptor superfamily, can inhibit the activation of NF-*κ*B through several mechanisms and repress the NF-*κ*B-mediated transcription of proinflammatory cytokines [122]. The intragastric administration of TSG (10, 30, and 60 mg/kg/day) for 7 days after colitis is induced by acetic acid irrigation dramatically attenuates acetic acid-induced colon lesions in mice, reverses body weight loss, and improves histopathological changes. TSG apparently decreases the content of MDA, which is a marker of lipid peroxidation. TSG appears to exert its beneficial effects on acetic acid-induced experimental colitis through the upregulation of the mRNA and protein levels of PPAR-*γ* and the inhibition of the NF-*κ*B pathway, which in turn decreases the protein overexpression of the downstream inflammatory mediators TNF-*α*, IL-6, and COX-2 [123].

Neuroinflammation is closely implicated in the pathogenesis of neurological diseases [124]. The hallmark of neuroinflammation is microglial activation. The activation of microglial cells and the consequent release of proinflammatory and cytotoxic factors, such as TNF-*α*, IL-1*β*, NO, and prostaglandin E synthase 2, possibly contribute to the onset of neurodegenerative diseases [125]. TSG suppresses matrix metalloproteinase expression and inflammation in rats with diet-induced atherosclerosis [102] and inhibits cyclooxygenase-2 activity and expression in RAW264.7 macrophage cells [126]. As a member of the intracellular phase II enzyme family, HO-1 is necessary to maintain cellular redox homeostasis. The overexpression of HO-1 markedly suppresses TNF-*α*, thereby inducing airway inflammation by the inhibition of oxidative stress [127]. TSG treatment strongly induces the expression of HO-1 in an NRF2-dependent manner. Furthermore, TSG attenuates the LPS-mediated activation of RAW264.7 cells and the secretion of proinflammatory cytokines, including IL-6 and TNF-*α* [128]. NADPH oxidase is recognized as a key ROS-producing enzyme during inflammation and widely expressed in various immune cells, such as macrophages, eosinophils, microglia, and neutrophils [129]. TSG attenuates LPS-induced NADPH oxidase activation and subsequent ROS production [130]. Therefore, TSG may be used to treat inflammatory diseases. However, further research should be performed.

## 6. Other Effects

Oxidative stress is believed to play a role in physiological and pathological aging processes, such as age-related neurodegenerative diseases. Various studies have been performed about the antiaging effects of TSG [2]. Klotho, a serum secretory protein, is closely related to age. Klotho has many physiological functions, such as regulating calcium and phosphorus levels in vivo, delaying senescence, improving cognition, reducing oxidative stress, and protecting vascular endothelial cells [131]. Klotho and insulin/IGF-1 signaling pathways are closely associated with ROS accumulation, and the protein levels of klotho and insulin signaling pathway are critical for antiaging [131]. The long-term administration of TSG improves the memory ability and regulates the body weight of mice with D-galactose-induced aging, reduces the levels of IGF-1, and increases the levels of klotho in serum. TSG upregulates the expression of klotho in the cerebrum, heart, kidney, testis, and epididymis tissues of mice with D-galactose-induced aging [132]. Further studies have shown that TSG extends the life span of mice by upregulating neural klotho and downregulating neural insulin or insulin-like growth factor 1 [3]. TSG enhances the stress resistance and increases the life span of the nematode *Caenorhabditis elegans* [1]. These results strongly confirm the potential of TSG as a pharmaceutical antiaging drug.

Cardiac fibroblasts play an important role in regulating normal myocardial function and adverse myocardial remodeling. Angiotensin (Ang) II, the effector peptide of the renin-angiotensin system, is a key pathogenic factor in the development of hypertension and heart failure. TSG can inhibit Ang II-induced cardiac fibroblast proliferation by suppressing the ERK1/2 pathway and reducing the overall production of extracellular matrix components [133]. TSG can prevent cardiac remodeling induced by pressure overload in rats. The underlying mechanisms may be related to a decreasing angiotensin II level, an antioxidant effect of the tested compound, the suppression of transforming growth factor-*β*1 expression, and the inhibition of ERK1/2 and p38 mitogen-activated protein kinase activation [134]. Further studies have suggested that the upregulation of endogenous PPAR-*γ* expression by TSG may be involved in the beneficial effect of TSG on pressure overload-induced cardiac fibrosis [17].

ROS can enhance bone resorption by directly or indirectly promoting osteoclast formation and activity [135]. TSG protects MC3T3-E1 cells from H_2_O_2_-induced cell damage and inhibition of osteoblastic differentiation. The protective effect of TSG on osteoblastic MC3T3-E1 cells may be mediated partly by its antioxidant ability [136]. In addition, TSG increases the density, content, and size of minerals in bone tissues and enhances the resistance to exogenic action, structural toughness, and strength of bone tissues [137]. These results suggest that TSG can be used as a good candidate for the protection of osteoblasts against oxidative stress-induced dysfunction and may have a potential therapeutic value for osteoporosis.

## 7. Conclusion

TSG has broad biological actions, including radical-scavenging effects and beneficial effects, for the treatment of various conditions, such as neuronal disease, cardiovascular disease, inflammatory disease, and osteoporosis. Several studies have elucidated the underlying mechanism of TSG action.

The effects of TSG against oxidative stress have helped elucidate many aspects of its mechanism of action. TSG is a strong free radical scavenger and potent antioxidant [24] (Table 1). TSG can enhance cognitive performance and prevent or treat AD in multistages and multitargets (Table 2). TSG may also act as an effective neuroprotective agent against PD through the modulation of the PI3K/Akt signaling pathway and ROS-mediated JNK, p38, and mitochondrial pathways [69–71, 80] (Table 3). TSG elicits anti-inflammatory effects and provides protection against cerebral I/R injury through multifunctional cytoprotective pathways (Tables 4 and 5). Previous studies demonstrated that TSG reduces the blood lipid content and inhibits the atherosclerotic process [102] (Table 6). TSG can prevent pressure overload-induced cardiac remodeling and Ang II-induced cardiac fibrosis in vitro [17] and may function against H_2_O_2_-induced dysfunction and oxidative stress in osteoblastic MC3T3-E1 cells (Table 7), but these abilities should be further investigated.

TSG has a wide variety of pharmaceutical properties. Its ameliorating effects and possible mechanisms should be summarized and determined. This review may provide a foundation for further studies to assess the mechanisms underlying the effects and clinical applications of TSG.

## Figures and Tables

**Table 1:** Antioxidative effect.

Experimental model	IC_50_/dose	Effects and possible mechanism	Reference number
	33.24 *μ*Μ	Scavenge DPPH radical	[24]
	2.75 and 0.57 *μ*g/ml	Scavenge ^·^OH and superoxide anion radical	[8]
HBMECs	50 and 100 *μ*M	↓ MDA and ROS; ↑ SOD and GSH	[25]
Rats	20 and 40 mg/kg	↑ SOD, GSH-Px activities; ↓ thiobarbituric acid reactive species content	[26]
Hypertensive rats	50 mg/kg	↓ Serum MDA and tissue 8-OHDG levels; ↑ serum GSH-Px activity	[27]
GES-1 cells and SGC-7901 cells	5 and 10 *μ*M	↓ ROS production; ↑ the expression of HO-1 and NQO1	[30]

**Table 2:** Protective effect against Alzheimer's disease.

Experimental model	Dose	Effects and possible mechanism	Reference number
SAMP8 mice	2, 20, and 50 *μ*M	Improves the memory ability and prolonged the life span of mice; ↑ the protein level of neural klotho; ↓ the levels of neural insulin, insulin-receptor, IGF-1, and IGF-1 receptor	[3]
SAMP8 mice	33, 100, and 300 mg/kg	Prevention of age-related learning and memory impairment; ↑ ChAT activity; ↓ AChE activity; ↑the synthesis of NE, DA, and 5-HT; ↓ glutamic acid and aspartic acid contents	[35–37]
APP695V717I transgenic mice	100 mg/kg	Improve learning, memory, and spatial orientation behavioral functions in mice; ↓ A*β* neurotoxicity-induced injury to endoplasmic reticulum functions	[41]
HEK-293FT cells; SH-SY5Y cells; APP/PS1 transgenic mice NaN_3_-induced rats	10–200 *μ*M; 50 mg/kg	Activation of AKT-GSK3*β* signaling pathway; ↓ APP–KPI^+^ and amyloid plaque formation; ↓ the splicing activity of ASF, APP–KPI^+^ levels, A*β* deposition	[15]
	60 and 120 mg/kg	↑ Mitochondrial COX activity; NGF, BDNF, TrkB expression; ↓ A*β*_1–42_ content; APP, BACE1, and PS1 expression	[42]
A*β*-induced N9 and BV2 cells	90 *μ*M	↓ iNOS, NO, COX-2, and PGE2, IL-1*β*, IL-6, and TNF-*α*; ↑ IL-10, BDNF, and GDNF, Arg-1; ↓ PU.1 overexpression	[44]
A*β*_1–42_-induced PC12 cells	0.1, 1, and 10 *μ*mol/l	↑ Cell survival rate; ↓LDH release, MDA content; ↑SOD activity and Bcl-2 expression	[47]
A*β*_1–40_-induced mice	100 mg/kg	Prevention of learning and memory impairment; ↓ IL-6 content, MDA content, and MAO-B activity; ↑ T-AOC activity	[48, 49]
A*β*_1–42_-induced mice	30, 60, and 120 mg/kg	↓ Oxidative damage; inhibited the expression of Keap1/Nrf2 pathway	[50]
A*β*-induced HT-22 cells	60 *μ*mol/l	↓ Oxidative stress; activation of Nrf2-HO-1 signaling	[51]
APPV717I transgenic mice	120 and 240 *μ*mol/kg	Improves learning–memory impairment; ↓ A*β* content; ↓ *α*-synuclein overexpression and aggregation	[40, 55]
Aged rats	30 and 60 mg/kg	↑ Synapses and synaptic vesicles, SYP expression in the hippocampal CA1 region	[56]
Aged mice	50,100, and 200 mg/kg	Enhances the memory and movement functions, protected the synaptic ultrastructure; ↑ synapse-related proteins and p-CaMKII	[10]
PC12 cells	1 *μ*M and 5 *μ*M	Induces neurite outgrowth, promotes PC12 cell differentiation; ↑ intracellular calcium levels, facilitates HFS-induced hippocampal LTP, activates CaMKII and extracellular ERK	[61]

**Table 3:** Protective effect against Parkinson's disease.

Experimental model	Dose	Effects and possible mechanism	Reference number
MPP^+^-induced PC12 cells	1–10 *μ*M	↑ Cell viability; ↓ cell apoptosis; ↓ intracellular ROS and the phosphorylated JNK; ↑ SOD, CAT, and GSH-Px activities, ↑ Bcl-2/Bax ratio, MMP; ↓ MDA content, cytochrome c, caspase-3; activation of PI3K/Akt pathway	[69–71]
MPP^+^-induced A53T AS cells	3.125–50 *μ*M	↓ *α*-Syn overexpression and aggregation; enhancing mitochondrial function; ↓ ROS level; ↓ cell apoptosis	[76]
6-OHDA-induced PC12 cells	10–50 *μ*M	↓ Intracellular ROS and NO; ↓ overexpression of iNOS, nNOS; ↓ 3-NT level	[79]
MPTP-induced mice	20 or 40 mg/kg	Reverses the MPTP-induced behavioral deficits; ↑ TH-positive neurons in SNpc ↑DA and its metabolites contents, and DAT protein in striatum; activation of PI3K/Akt pathway; inhibition of the ROS-mediated JNK, P38, and mitochondrial pathways	[16, 80]
6-OHDA-induced rats; primary rat midbrain neuron-glia cocultures	10 and 50 mg/kg; 20–80 *μ*M	↓ Neurotoxicity; suppressed microglia activation and proinflammatory factors; inactivation of MAPK signaling pathway	[83]
Primary rat microglia- and astroglia-enriched cultures; LPS-induced rats	20–80 *μ*M; 10 and 50 mg/kg	↓ Microglia-mediated neuroinflammation; enhancing astroglia-derived neurotrophic effects	[11]

**Table 4:** Protective effects against cerebral ischemia injury.

Experimental model	Dose	Effects and possible mechanism	Reference number
OGD-R-induced cell; MCAO mice	25 *μ*M; 15 and 40 mg/kg	↓ Neuronal injury; ↓ intracellular ROS; ↓ [Ca^2+^]_i_, ↑ MMP; ↓ brain infarct volume; ↓ cell apoptosis	[18]
Rat	0.038, 0.114, and 0.342 g/kg	↑ GSH-P_X_ and SOD activities; ↓ MDA content	[88]
Gerbils	0.038, 0.114, and 0.342 g/kg	↓ Binding force of NMDA receptor; ↓ intracellular [Ca^2+^]_i_	[89]
Rats	60 mg/kg	↑ The protein expression of HIF-1*α* and EPO	[91]
Rats	30, 60, and 120 mg/kg	↓ Cell apoptosis; ↑ Bcl-2; ↓ Bax	[92]
Rats	30, 60, and 120 mg/kg	↓ AChE activity; ↑ the expression of protein PP-2A and MAP-2	[95]
Rats	30, 60, and 120 mg/kg	Promoted postoperative recovery in rats; ↓ volume of cerebral infarcts; improving neurological dysfunction; ↑ microvessel density in the brain; ↑ CD31 expression; ↑ levels of VEGF, Ang 1, and Tie-2	[12]

**Table 5:** Effects on inflammatory disease.

Experimental model	Dose	Effects and possible mechanism	Reference number
Mice	10, 30, and 60 mg/kg	Attenuates acetic acid-induced colon lesions; reverses body weight loss, and improves histopathological changes; ↓ the content of MDA; ↑ the mRNA and protein levels of PPAR-*γ*; inhibition of the NF-*κ*B pathway; ↓ the protein overexpression of the TNF-*α*, IL-6, and COX-2	[123]
RAW264.7 macrophage cells	1, 10, and 100 *μ*mol/l	↓ COX-2 enzyme activity and expression	[126]
Microglia BV2 cell lines	20–80 *μ*M	↓ NADPH oxidase activation; ↓ ROS production	[130]

**Table 6:** Antiatherosclerosis effect.

Experimental model	Dose	Effects and possible mechanism	Reference number
Hyperlipidemic rats	90 and 180 mg/kg	↓ Serum TC, LDL-C, and AI levels; ↑ the mRNA expression of LDLR in the liver cells	[100]
Hyperlipidemia rats	12 and 24 g/kg	↓ Serum TC, TG, LDL-c, apoB, and MDA; ↑ serum SOD, CAT, GSH-P_X_, and T-AOC; ↑ the ratios of HDL-c/TC and apoAI/apoB	[101]
ApoE^−/−^ mice; RAW264.7 cells	50 and 100 mg/kg; 10 and 100 *μ*M	↓ TC, TG, and LDL-C; ↑ HDL-c, SR-BI, ABCG5, and CYP7A1 expression; ↑ the protein expressions of ABCA1 and ABCG1	[104]
Atherosclerotic rats	30, 60, and 120 mg/kg	↑ NO levels in the serum and aorta; ↑ NOS content and the expression of eNOS, eNOS mRNA; ↓ the expression of iNOS, iNOS mRNA	[103, 107]
Atherosclerotic rats	120, 60, or 30 mg/kg	↓ CRP, IL-6, and TNF-*α* levels	[102]
HUVECs	0.1, 1, or 10 *μ*mol/l	↓ Mitochondrial apoptotic pathway, lipid peroxidation, ROS, and MDA; ↑ SOD and GSH-Px; ↓ the expression of Notch1, Hes1, and MCP-1	[112, 113]
VSMCs	10 and 100 *μ*mol/l	↓ VSMC proliferation; ↓ cell cycle transition from G0/G1 phase to S phase; ↓ PCNA expression in the nucleus of VSMCs	[116]
HUVECs	50 and 100 *μ*M	↓ Vimentin mRNA and protein levels; ↓ TGF*β*/Smad signaling pathway and caspase-3 activation	[119]

**Table 7:** Other effects.

Experimental model	Dose	Effects and possible mechanism	Reference number
D-galactose-induced aging mice	42, 84, and 168 mg/kg	Improves the memory ability and regulates the body weight of mice; ↓ the levels of IGF-1; ↑ the expression of klotho	[132]
*C. elegans*	50 and 100 *μ*M	Enhances the stress resistance; ↑ the life span of the nematode *C. elegans*	[1]
Rat cardiac fibroblasts	3–100 *μ*mol/l	↓ ERK1/2 activation; ↓ overall production of ECM components	[133]
Pressure overload rats	60 and 120 mg/kg	↓ Angiotensin II level; ↓ transforming growth factor-*β*1 expression; ↓ ERK1/2 and p38 MAPK activation	[134]
Pressure overload rats	120 mg/kg	↑ Endogenous PPAR-*γ* expression	[17]
MC3T3-E1 cells	0.1–10 *μ*M	↓ Osteoblastic differentiation; ↓ oxidative damage	[136]
SD rats	150, 300, and 600 mg/kg	↑ The density, content, and size of minerals in bone tissues; enhances the resistance to exogenic action, structural toughness, and strength of bone tissues	[137]

## Abbreviations

PM: *Polygonum multiflorum* Thunb
TSG: 2,3,5,4′-Tetrahydroxystilbene-2-*O*-*β*-D-glucoside
ROS: Reactive oxygen species
H_2_O_2_: Hydrogen peroxide
SOD: Superoxide dismutase
GSH-Px: Glutathione peroxidase
TBARS: Thiobarbituric acid reactive species
HUVECs: Human umbilical vein endothelial cells
HO-1: Hemeoxygenase-1
NQO1: NADPH-quinone oxidoreductase 1
A*β*: Beta-amyloid peptide
APP: Amyloid precursor protein
ChAT: Acetylcholine transferase
ACH: Acetylcholine
SAMP8: Senescence-accelerated-prone 8
IGF-1: Insulin-like growth factor-1
KPI: Kunitz protease inhibitor
BACE1: Beta-site APP cleaving enzyme 1
PS1: Presenilin 1
TrkB: Tropomyosin-related kinase B
SYP: Synaptophysin
NMDA: *N*-Methyl-D-aspartate
CaMKII: Calcium–calmodulin-dependent protein kinase II
ERK: Extracellular signaling-regulated kinase
IL-6: Interleukin-6
MPTP: 1-Methyl-4-phenyl-1,2,3,6-tetrahydropyridine
MPP^+^: 1-Methyl-4-phenylpyridinium
MDA: Malondialdehyde
Bax: Bcl2-associated protein
Bcl-2: B-cell lymphoma protein-2
MCAO: Middle cerebral artery occlusion
MAP-2: Microtubule-associated protein-2
PP-2A: Protein phosphatase-2A
VSMC: Vascular smooth muscle cell
LDL: Low-density lipoprotein
LDL-C: Low-density lipoprotein cholesterol
NO: Nitric oxide
iNOS: Inducible NO synthase
TNF-*α*: Tumor necrosis factor
CRP: C-reactive protein
PCNA: Proliferating cell nuclear antigen
COX: Cyclooxygenase.
